# Supplementary material for: The Construction and Exploration of a Comprehensive MicroRNA Centered Regulatory Network in Foxtail Millet (Setaria italica L.)
Source: Front Plant Sci. 2022 May 6;13:848474. doi: 10.3389/fpls.2022.848474 (PMC9121102; doi:10.3389/fpls.2022.848474)
Supplement: Supplementary file 2 [file Data_Sheet_2.zip › Sit-miR168_Seita.3G070600.1_747_TPlot.pdf]

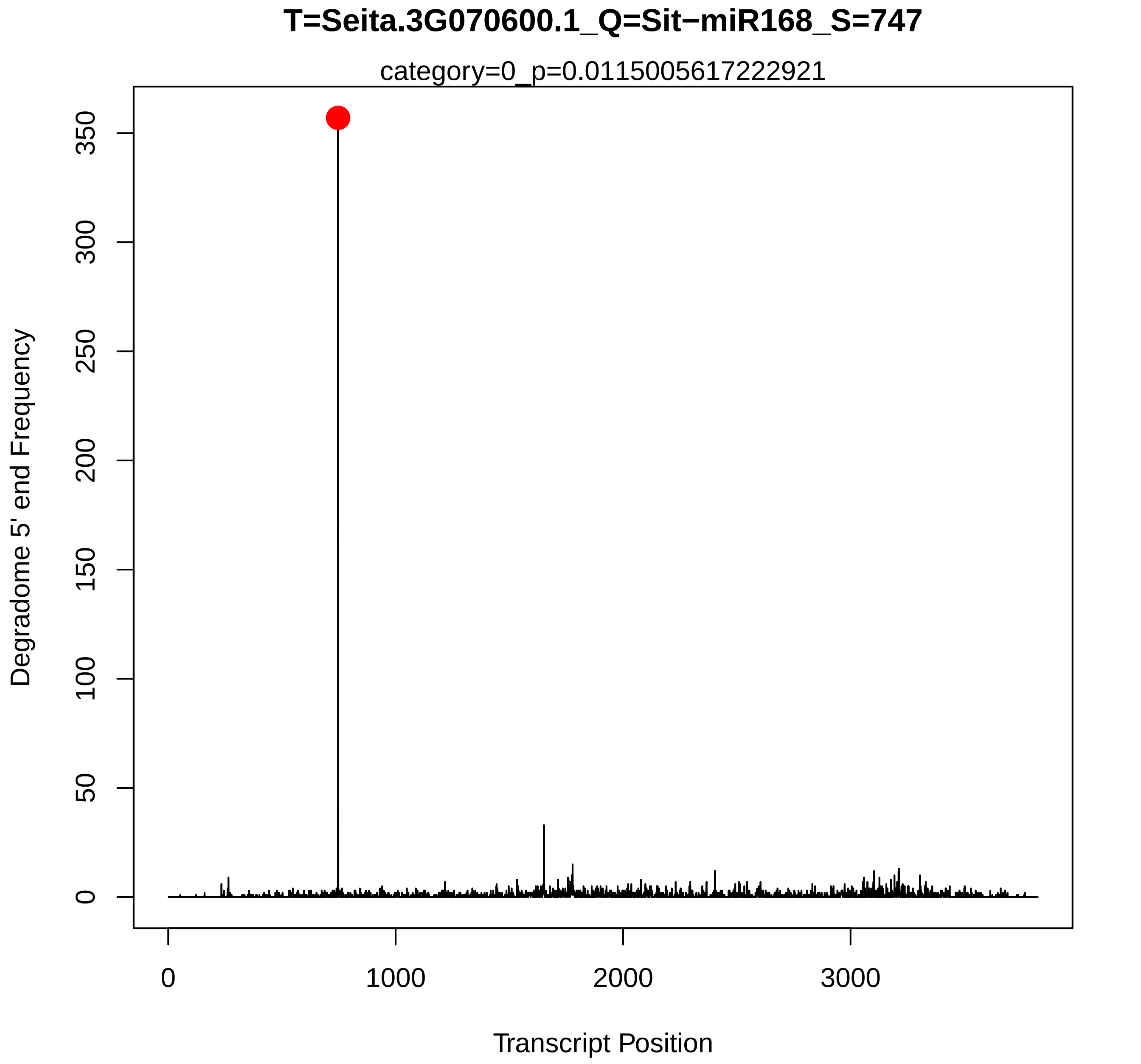

T=Seita.3G070600.1_Q=Sit−miR168_S=747
category=0_p=0.0115005617222921
Degradome 5' end Frequency
0
50
100
150
200
250
300
350
0
1000
2000
3000
Transcript Position
